# *iotbx.cif*: a comprehensive CIF toolbox

**DOI:** 10.1107/S0021889811041161

**Published:** 2011-10-29

**Authors:** Richard J. Gildea, Luc J. Bourhis, Oleg V. Dolomanov, Ralf W. Grosse-Kunstleve, Horst Puschmann, Paul D. Adams, Judith A. K. Howard

**Affiliations:** a Durham University, Department of Chemistry, Durham DH1 3LE, UK; b Lawrence Berkeley National Laboratory, Physical Biosciences Division, MS 64R0121, CA 94720, USA

**Keywords:** *iotbx.cif*, *cctbx*, CIF, computer programs

## Abstract

*iotbx.cif* is a new software module for the development of applications that make use of the CIF format. Comprehensive tools are provided for input, output and validation of CIFs, as well as for interconversion with high-level *cctbx* [Grosse-Kunstleve, Sauter, Moriarty & Adams (2002). *J. Appl. Cryst.* **35**, 126–136] crystallographic objects. The interface to the library is written in Python, whilst parsing is carried out using a compiled parser, combining the performance of a compiled language (C++) with the benefits of using an interpreted language.

## Abstract 2

*iotbx.cif* is a comprehensive toolbox for the development of applications that make use of the CIF format.

## 1. Introduction

The CIF (Crystallographic Information File) syntax (Hall *et al.*, 1991 ▶) has become firmly established (Brown & McMahon, 2002 ▶; Spek, 2009 ▶) as the file format for deposition and archiving of small-mol­ecule crystal structures and increasingly their structure factors. Whilst the Protein Data Bank (PDB) format (http://www.wwpdb.org) is still the prevailing file format for deposition of macromolecular crystal structures, the CIF format is nonetheless important to macromolecular software through their extensive use of the PDB chemical components (http://www.wwpdb.org/ccd.html) and CCP4 monomer libraries (Vagin *et al.*, 2004 ▶).

In addition to the core CIF dictionary (Hall, Allen & Brown, 2005 ▶), the International Union of Crystallography (IUCr) maintains CIF dictionaries for describing the results of macromolecular (Fitzgerald *et al.*, 2005 ▶), powder diffraction (Toby, 2005 ▶) and electron density studies (Mallinson & Brown, 2005 ▶), and for describing incommensurately modulated crystal structures (Madariaga, 2005 ▶). The Crystallography Binary Format (CBF) and image-supporting Crystallographic Information File (imgCIF) (Bernstein & Hammersley, 2005 ▶) are extensions to the CIF format to support inclusion of binary data in the CIF, in particular raw experimental data from area detectors. Furthermore, CIF is probably one of the most well known file formats within the field of chemistry, since it is predominantly the form in which chemists receive the results of a crystal structure analysis carried out on their behalf.

The CIF format is intrinsically involved in a wide variety of crystallographic applications from data collection to publication and archiving of the outcomes of crystallographic studies. In addition there is a wealth of crystal structure coordinates and reflection data freely available in CIF format through the Crystallographic Open Database (COD; Gražulis *et al.*, 2009 ▶) and the large quantity of data available as supplementary material for papers published in IUCr journals, for which many possible uses can be imagined. As such it is vital for a crystallographic library such as the *cctbx* (Grosse-Kunstleve *et al.*, 2002 ▶) to provide high-quality tools for reading, creation and manipulation of CIFs, and extraction of crystallographic information from them.

Several CIF programming libraries have been developed for various languages and environments, including Fortran (Hall & Bernstein, 1996 ▶; Rodriguez-Carvajal & González-Platas, 2003 ▶), C (Ellis & Bernstein, 2001 ▶; Westbrook *et al.*, 1997 ▶), Objective C (Chang & Bourne, 1998 ▶), .NET (Lin, 2010 ▶), Java (Day *et al.*, 2011 ▶), Perl (Bluhm, 2000 ▶) and Python (Hester, 2006 ▶). Whilst there existed several partial CIF parsers within the *cctbx*, each hand-crafted to suit a specific task [separate tools for reading the PDB chemical components and CCP4 monomer libraries (Painter & Merritt, 2004 ▶); as part of the phenix.cif_as_mtz tool; for reading fcf reflection files as output by *SHELXL* (Sheldrick, 2008 ▶)], a comprehensive CIF parser that was tightly integrated with the rest of the library was conspicuously absent.

During the development of the *smtbx* (small-molecule toolbox) and *OLEX2* (Dolomanov *et al.*, 2009 ▶), it became apparent that the CIF format would play a central part in presenting the results of the procedures developed. In addition, there was a need to provide an interface for managing the contents of the CIF within *OLEX2*. Therefore it was decided to implement a new CIF framework within the *iotbx* (input/output toolbox) module of the *cctbx*.

Given the availability of a clearly defined formal grammar for the CIF syntax (Hall, Spadaccini *et al.*, 2005 ▶), it was decided to use the ANTLR parser generator (Parr, 2007 ▶) for the automatic generation of a lexer and parser. ANTLR was chosen because of its support for multiple programming languages, in particular its support for Python and C/C++. In addition, the associated ANTLRWorks (http://www.antlr.org/works/index.html) GUI development environment features a number of tools that aid the development of grammars, including visualization of syntax diagrams and rule dependency graphs. This enabled the majority of the development to be focused on the design of the internal representation of the CIF model, whilst ensuring that the resulting parser closely follows the formal CIF grammar. The code is structured in such a way that the parser is quite distinct from the model, meaning that an alternative representation of the model could be used with the same parser, and conversely a different parser could be used to populate the existing *iotbx.cif* model.

As is standard practice in the *cctbx*, all aspects of the code are rigorously tested by unit tests, guarding against future regression of the code base. In addition the unit tests provide usage examples for the programmer interface.

## 2. Validation of CIFs against data dictionaries

Successful parsing without errors of a given CIF indicates only that it is syntactically correct. CIF dictionaries allow for a machine-readable formal description of allowed data items and for possible restrictions on the attributes of their associated values. A collection of application-specific dictionaries are maintained by the Committee for the Maintenance of the CIF Standard (COMCIFS), and can be used to validate the contents of a given CIF. The CIF data dictionaries abide by the CIF syntax, with two distinct dictionary definition languages [DDL1 (Hall & Cook, 2005 ▶) and DDL2 (Westbrook *et al.*, 2005 ▶)] currently in use.

In the context of *iotbx.cif*, a CIF can be validated as follows:
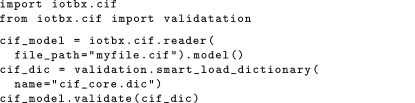


The smart_load_dictionary function allows for a dictionary to be loaded from a variety of sources, including from a locally stored version, by downloading from an arbitrary URL or *via* lookup in a CIF dictionary register (*e.g.* ftp://ftp.iucr.org/pub/cifdics/cifdic.register), allowing use of the most up-to-date version of the dictionary. A list of potential errors and warnings found during the validation is output by the procedure. The error handling is designed such that it is possible for an application making use of *iotbx.cif* to override the default error handler with one specific to the needs of the application.

## 3. Common CIF syntax errors and error recovery

As a result of comprehensive testing of the *iotbx.cif* parser a number of commonly encountered syntax errors were identified. Among the sources of CIFs used are the COD, a selection of CIFs obtained from the IUCr journals web site, the PDB chemical components library, the CCP4 monomer library and the Durham in-house database of crystal structures. Among the commonly found errors are the following:

(1) Missing starting and closing quotes.

(2) Missing starting and closing quotes for a string containing whitespace.

(3) Some text prepended to the CIF but not using CIF comment format.

(4) Mismatching semicolon delimiters for a multi-line text field.

(5) More than one data value per tag.

(6) Missing data value for a tag.

(7) Incomplete CIF – *e.g.* missing data block heading.

(8) Intended data block heading containing whitespace or illegal character(s). This can happen if a program uses the file name as the data block heading when creating a CIF but does not remove/replace whitespace or illegal characters.

(9) Non-ASCII characters – data values have been copied from other sources; for example, this could be an author’s name or a place name.

(10) Unquoted string with ‘[’ as the first character.

(11) Wrong number of values for a loop.

(12) Inclusion of an unnamed global_ block.

Item (10) was the syntax error most commonly observed in the publicly available databases (*i.e.* excluding the Durham in-house database), possibly because common syntax checking routines do not currently flag this as an error. The CIF grammar explicitly forbids the characters ‘[’ and ‘]’ from being the first character of an unquoted string (Hall, Spadaccini *et al.*, 2005 ▶).

The inclusion of unnamed global_ data blocks, whilst allowed by the STAR grammar (Hall & Spadaccini, 1994 ▶), is expressly forbidden by the current version of the CIF grammar, with the case-insensitive word global listed as a reserved word that may not appear as an unquoted data value in a CIF. However, in order to support parsing of the CCP4 monomer library with *iotbx.cif*, a non-strict parsing mode was added which permits the presence of global_ data blocks.

The most commonly encountered syntax error for CIF format reflection files is item (11), although this error can affect any CIF containing looped data items. The number of values in a loop must be an exact multiple of the number of tags in the loop header and it is an error if this is not the case. This is probably the hardest error to diagnose since it is not associated with a specific line number, only the particular loop, which may be many thousands of lines long in the case of reflection data, and hence the entire loop is rendered invalid. Frequently this error can be attributed to manual editing of the file resulting in one or more values being accidentally deleted or inserted. More worryingly, it is occasionally the result of a program outputting the data in fixed-field format when one of the values takes up the full width of its fixed field, losing a whitespace separator in the process.

Some of the syntax errors outlined above are, to varying degrees, recoverable parsing or lexing errors. Missing quotes potentially can be detected and missing tokens inserted when an end-of-line (EOL) character is encountered, since a quoted string cannot extend past an EOL character. For errors such as multiple values for a tag that is not part of a loop, or a tag with no value given, the parser may recommence parsing at the next valid token it finds, discarding those invalid tokens. For invalid characters [items (9) and (10)], either the invalid characters can be accepted or the offending tag–value pair can be discarded (the current implementation does the latter). The most problematic error is that of a missing closing semicolon for a semicolon text field, since the rest of the file up to the end-of-file (EOF) character is consumed as part of the semicolon text field. Upon reaching the EOF character an error is emitted by the lexer, but automatic recovery from this error is not possible.

On the one hand, it may be desirable for a program to be as accommodating of errors as possible on input whilst ensuring that the output is as correct as possible. On the other hand, there are clear advantages in having software that raises informative errors when syntax errors are encountered, as this would discourage the proliferation of incorrectly formed CIFs.

*iotbx.cif* provides two modes for error handling: in the strict mode an exception is raised if any errors are encountered during parsing; in the non-strict mode parsing continues after recovery from any errors, providing a list of all errors encountered for examination.

## 4. Using *iotbx.cif*

Developers familiar with the built-in dictionary type of the Python programming language (Python Software Foundation, 2011 ▶) will be immediately at home with the syntax of the *iotbx.cif* representation of the CIF model.

The top-level object is iotbx.cif.model.cif, which is the type equivalent to a full CIF file. This contains zero or more data blocks, which are accessed by data block name using the standard Python dictionary square brackets notation for accessing a dictionary by key. Using a valid data block name, this returns a CIF data block of the type iotbx.cif.model.block. A CIF data block consists of a sequence of data items and associated values. A data item can be associated with either one value or a list of values (as part of a CIF loop), and a given data item can only be found once per data block. These values can in turn be accessed using the square bracket notation to retrieve the value(s) associated with a specified data item (tag):
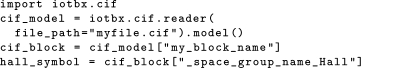


then
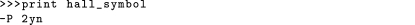


Looped items are stored by column, and the full list for a given looped item can be accessed by the data name as shown below:
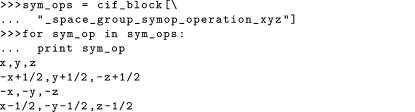


The full loop object can be extracted *via* the name of the loop. The name of the loop is taken to be the longest common substring starting with an underscore character, and followed by (but not including) an underscore (in the case of DDL1-compliant CIFs) or point (in the case of DDL2-compliant CIFs) character separator. This follows the IUCr guidelines for reserved prefixes for local dictionary extensions (http://www.iucr.org/resources/cif/spec/ancillary/reserved-prefixes). Once a loop has been extracted, this can then be used to iterate through by row, or to add further rows or columns to the loop. The following example demonstrates the creation of a CIF loop containing the symmetry operations of a given space group:
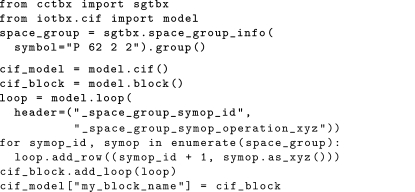


CIF objects (model.cif, model.block, model.loop) can be exported in CIF format in several ways. The simplest way is using the Python print statement as follows:
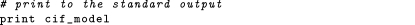


 or alternatively
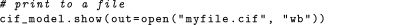


The show() method of the CIF object allows more fine tuning of the output, including the amount of indentation used for looped data and the width of the data name field. For more advanced formatting, a Python formatting string can be provided to control the output of individual loops (in contrast to the default behaviour where items are single space separated).

Further documentation and example code can be found at http://cctbx.sourceforge.net/iotbx_cif/.

### 4.1. Interconversion with *cctbx* crystallographic objects

An essential part of any crystallographic library or software is a means to easily export/extract crystallographic information to/from common crystallographic file formats. As such, two central crystallographic objects in the *cctbx*, namely xray.structure and miller.array, have methods enabling easy interconversion of either object with a CIF. The xray.structure class comprises the objects needed for calculation of structure factors – scatterers, site symmetry, crystal symmetry – and provides many methods for manipulating the structure. Similarly, the miller.array class brings together a set of miller indices and associated data – such as intensities or amplitudes, complex structure factors or *R*_free_ flags – along with relevant methods for acting on that data.

All crystal structures and miller arrays can be extracted from a given CIF as follows:
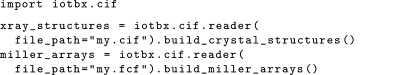


Tools have been developed in order to support output of the requisite structural information for publication of a structure determination. This includes the export of an xray.structure to CIF format, and also the inclusion of geometrical features such as bonds and angles. Optionally the covariance matrices for the refined parameters and the unit-cell parameters can be provided to enable the calculation of standard uncertainties for both refined and derived parameters.

*iotbx.cif* also includes support for the recently introduced restraints CIF dictionary (ftp://ftp.iucr.org/pub/cif_core_restraints.dic), which is intended to allow for the description in CIF format of the restraints and constraints used in a least-squares refinement.

## 5. Performance

Whilst for many applications the processing of input files is not usually a time-critical part of the program, it is important that the overhead of file processing is minimal when using batch processing of large numbers of files, in particular during the development and testing of new algorithms or curation of ever-expanding CIF databases. Since the program uses a compiled (C++) parser, it is expected that parsing would be of sufficient speed to make handling even of large CIF files interactive, including processing of files containing structure factors. To test the performance of the parser and the procedures for extracting crystallographic information, a short script was run over all the CIF files in the COD, using an Apple MacBook Pro with Quad-core Intel Core i7 (2.2 GHz).

A total of 145 559 CIFs were parsed at an average of 13 ms per file, with only one file found to contain syntax errors; the remainder all parsed successfully. The procedure was repeated in order to construct instances of xray.structure; a total of 143 882 instances were successfully constructed at an average of 23 ms per file. When the procedure was run over 14 420 CIF-format reflection files found in the COD, the average parsing time for a reflection file was 83 ms, increasing to 115 ms when construction of a miller.array was attempted after parsing. Table 1 ▶ gives the performance of *iotbx.cif* tools on typical small-molecule and protein data files and two selected dictionary files.

The results show good performance for both the parser and the procedures for extracting crystallographic information from the CIF model. With the increasing availability of multi-core processors, it is clear that, in conjunction with the large number of tools provided by the *cctbx*, *iotbx.cif* is suitable for performing large-scale analyses of crystal structures, since the overhead of reading structures from CIFs is minimal.

The performance of CIF output for files containing loops with a large number of values can be improved significantly by using the advanced loop formatting option described in §4, since each value will no longer be checked individually to determine if quoting of the value is necessary.

As a by-product of the comprehensive testing of *iotbx.cif* on the COD, numerous syntactic and semantic errors were identified and communicated to the curators of the database. Using current hardware with 48 processor cores, it is possible to run *iotbx.cif* over the entirety of the COD (>160 000 files at the time of writing) in little over two minutes, demonstrating the potential use of *iotbx.cif* to aid the curation of CIF databases.

## 6. Conclusion

The *iotbx.cif* module is now used heavily by the *OLEX2* (Dolomanov *et al.*, 2009 ▶) and *PHENIX* (Adams *et al.*, 2010 ▶) software packages. Additionally, the tools provided by the *iotbx.cif* module are currently being used extensively, in conjunction with the COD as a source of structural models and associated reflection data, in the evaluation of different approaches to minimization (Grosse-Kunstleve, 2011 ▶).

With the addition of the *iotbx.cif* module, the *cctbx* now comprehensively supports most major small-molecule and macromolecular crystallographic file formats [*SHELX* ins/res and hkl, CIF, PDB, CCP4 maps (http://www.ccp4.ac.uk/html/maplib.html), *X-PLOR* hkl and map (Brünger, 1993 ▶), and MTZ format (http://www.ccp4.ac.uk/html/mtzformat.html) among others].

We believe that one of the strengths of *iotbx.cif*, and one that distinguishes it from similar software libraries, is its tight integration with the *cctbx* and higher-level crystallographic objects, providing immediate access to a wealth of crystallographic tools. *iotbx.cif* is equally suitable for integration into large-scale applications, stand­alone scripts or batch processing of large numbers of CIFs.

## 7. Availability

*iotbx.cif* is available as part of the *cctbx*, which is released under a nonrestrictive open-source licence. Source code and links to precompiled binaries for a large number of Windows, Macintosh and Linux systems can be found at http://cctbx.sourceforge.net/.

## Figures and Tables

**Table 1:** Execution times on (*a*) an Intel Xeon Processor E5520 (2.27 GHz) running Microsoft Windows 7 Ultimate and (*b*) an Apple MacBook Pro with Quad-core Intel Core i7 (2.2 GHz)

		Read time (ms)		Write time (ms)		Validation time (ms)	
File name†	File size (kB)	(*a*)	(*b*)	(*a*)	(*b*)	(*a*)	(*b*)
fg3210sup1.cif	25	25	21	20	18	39	28
fg3210cpsup2.hkl	84	42	32	131	106	42	34
4hhb.cif	705	727	604	2061	1622	2658	1312
cif_core_2.4.2.dic	476	142	116	84	69	236	192
mmcif_std_2.0.09.dic	1716	788	677	368	298	1596	1313

† 4HHB.cif (Fermi *et al.*, 1984 ▶) was downloaded from the PDB web site; fg3210sup1.cif and fg3210CPsup2.hkl (Yufit & Howard, 2011 ▶) were obtained from the IUCr web site.
